# Intraoperative Pancreatoscopy During Robotic Pancreatoduodenectomy and Robotic Distal Pancreatectomy for Intraductal Papillary Mucinous Neoplasm with Involvement of the Main Pancreatic Duct

**DOI:** 10.1097/AS9.0000000000000283

**Published:** 2023-05-05

**Authors:** Zhi Ven Fong, Maurice J.W. Zwart, Myrte Gorris, Rogier P. Voermans, Roy L.J. van Wanrooij, Thijs Wielenga, Marco del Chiaro, Urban Arnelo, Freek Daams, Olivier R. Busch, Marc G. Besselink

**Affiliations:** From the * Department of Surgery, Amsterdam UMC, Location University of Amsterdam, Amsterdam, the Netherlands; † Dana Farber Cancer Institute, Mass General Brigham, Harvard Medical School, Boston, MA; ‡ Cancer Center Amsterdam, the Netherlands; § Department of Gastroenterology and Hepatology, Amsterdam UMC, Location University of Amsterdam, Amsterdam, the Netherlands; ∥ Department of Gastroenterology and Hepatology, Amsterdam UMC, Location Vrije Universiteit, Amsterdam, the Netherlands; ¶ University of Colorado, CU Anschutz Medical Campus, Aurora, CO; # Department of Surgical and Perioperative Sciences, Surgery, Umeå University, Umeå, Sweden; ** Department of Surgery, Amsterdam UMC, Location Vrije Universiteit, Amsterdam, the Netherlands.

**Keywords:** Pancreas, surgery, minimally invasive, robotic, pancreatoscopy, intraductal papillary mucinous neoplasm

## Abstract

**Background:**

Intraductal papillary mucinous neoplasm (IPMN) with involvement of the main pancreatic duct usually requires surgical resection. Consensus is lacking whether to partially or completely resect the pancreatic portion with a dilated main pancreatic duct. Intraoperative pancreatoscopy may be useful to determine the extent of IPMN to tailor surgical resection and was recently studied in a large prospective international study. IPMN is increasingly utilized using a robotic approach. Studies describing the technical approach to intraoperative pancreatoscopy in robotic pancreatoduodenectomy and robotic distal pancreatectomy are lacking.

**Methods:**

During robotic pancreatoduodenectomy, pancreatoscopy is performed once the pancreas neck is transected. The scope is advanced via a laparoscopic port into the left and right-sided pancreatic duct, guided by robotic graspers. During robotic distal pancreatectomy, pancreatoscopy is performed before complete parenchymal transection. The scope is advanced through an anterior ductotomy to examine the duct and guide the pancreatic transection line. Tips and tricks how to perform the procedure efficiently without complications are detailed.

**Results:**

In total, 28 robot-assisted pancreatoscopies were performed during robotic pancreatoduodenectomy and robotic distal pancreatectomy. No intraoperative complications resulting from the intraoperative pancreatoscopy were noted. In the 2 described procedures, the added time required to perform the pancreatoscopy was 6 and 17 minutes, respectively. Both patients recovered without complication and were discharged on postoperative day 5 for the robotic pancreatoduodenectomy and day 6 for the robotic distal pancreatectomy.

**Conclusions:**

Intraoperative pancreatoscopy can be safely performed during both robotic pancreatoduodenectomy and robotic distal pancreatectomy for IPMN with the involvement of the main pancreatic duct. An international prospective study has recently been completed with this technique.

## INTRODUCTION

Intraductal papillary mucinous neoplasm (IPMN) of the pancreas are intraductal lesions of the pancreas with a histologic spectrum ranging from benign to invasive ductal carcinoma.^1,2^ With the advances in cross-sectional imaging and its increased utilization, IPMNs have become increasingly common, often presenting as incidentally found pancreatic ductal dilatation. Patients at low risk of malignancy are observed with interval imaging, whereas patients with higher-risk lesions are recommended to undergo surgical resection if the patient is a good surgical candidate.^2^ Although high-quality imaging can accurately identify IPMNs, it is less helpful to pancreatic surgeons when trying to determine the extent of the resection intraoperatively. Peroral pancreatoscopy allows for ductal evaluation but also does not allow for marking to guide intraoperative decision-making, and is associated with a risk of postprocedural pancreatitis of up to 17%.^3^

Intraoperative pancreatoscopy has emerged as a safe approach that allows for direct visualization of IPMN in the main pancreatic duct and may thus tailor intraoperative decision-making. Based on retrospective studies, intraoperative pancreatoscopy findings changed the resection in approximately 17–22% of main-duct IPMN resections.^4,5^ Intraoperative pancreatoscopy has traditionally been performed with a flexible endoscope through a laparotomy incision during an open pancreatic resection. However, pancreatectomies for lesions such as IPMN are increasingly being performed robotically,^6–8^ without publications on pancreatoscopy during robotic pancreatoduodenectomy and robotic distal pancreatectomy. Herein, we detail our technical approach on intraoperative pancreatoscopy during robotic pancreatoduodenectomy and distal pancreatectomy for main-duct IPMN.

## METHODS

### Personnel and Equipment

Although the pancreatoscopy is performed by pancreatic surgeons we collaborate with dedicated gastroenterologists to allow for intraoperative multidisciplinary interpretation of the findings as gastroenterologists bring added experience of ductal evaluation.^3^ For the intraoperative application of pancreatoscopy, we utilize the SpyGlass Direct Visualization System (Boston Scientific, Natick, MA), and the SpyBite Forceps (Boston Scientific) when a biopsy is required. The diameter of the pancreatoscope is 10 Fr (3.33 mm) with a 1.2 mm therapeutic channel for biopsy purposes and can be easily passed through any 5 mm or wider port. We use the DaVinci Xi robotic system (Intuitive Surgical, Sunnyvale, CA).

### Pancreatoscopy During Robotic Pancreatoduodenectomy

**FIGURE 1.**
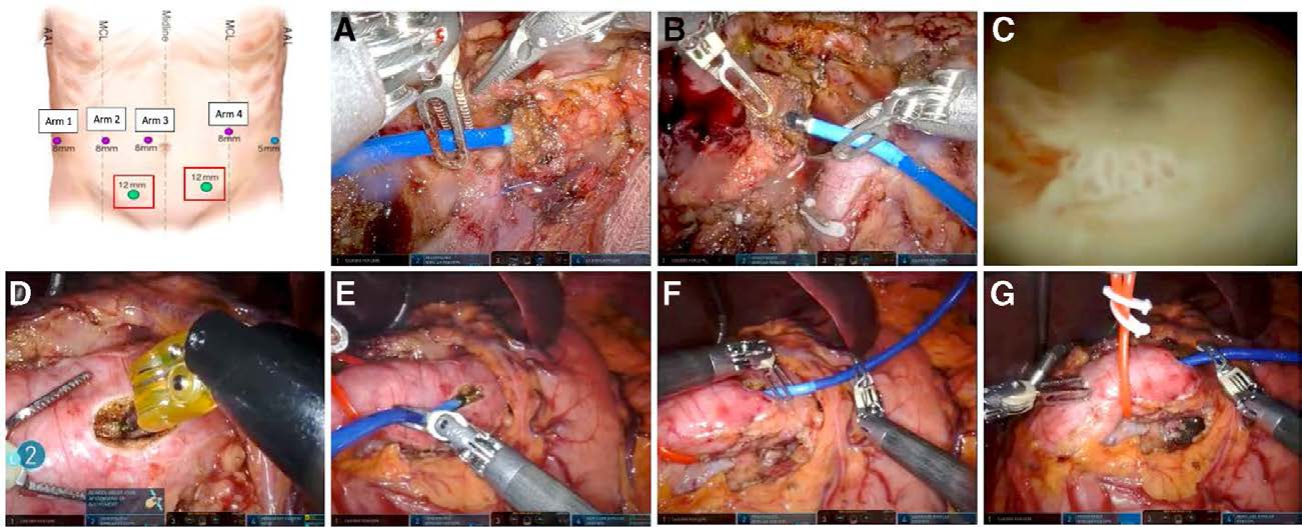
Port placement during robotic pancreatectomy, and procedural steps for performing intraoperative pancreatoscopy during robotic pancreatoduodenectomy (A–C) and robotic distal pancreatectomy (D–G). A, pancreatoscope is guided into the pancreatic duct of the tail gland with the left cadiere forceps whereas the right cadiere forceps gently grasp the peripancreatic tissue for counter-traction. B, the head gland is similarly inspected with both sides mobilized off the portal and superior mesenteric vein to avoid traction injury. C, intraductal view through the pancreatoscope demonstrating a dilated pancreatic duct with papillary projections. D, ductotomy performed with a hook cautery. E, pancreatoscope introduced to the distal gland entering the duct from the abdomen’s right and with counter-traction being provided with the vessel loop around the gland. F, pancreatoscope introduced to the proximal gland entering the duct from the abdomen’s left with both cardiere forceps facilitating its entry. G, bipolar forceps used to delineate the line of pancreatic transection based on intraductal findings on the pancreatoscope.

The port placement for our robotic pancreatic resections is depicted in Fig. 1. During a robotic pancreatoduodenectomy, the pancreatoscopy is performed after the division of the pancreatic neck. Both sides of the pancreas gland are mobilized for 1 cm off the portal and superior mesenteric vein to allow for gland manipulation while minimizing the risk of traction injury during pancreatoscopy. The pancreatoscope is introduced into the abdomen via either one of the 5–12 mm assistant laparoscopic ports. Two robotic cadiere forceps are used to guide the pancreatoscope into the pancreatic duct, with one grasper guiding the pancreatoscope and the other grasping on the peripancreatic tissue for counter-traction. It is important to note that while the SpyGlass Direct Visualization System is a single-use instrument, the robotic cadiere forceps has a risk of damaging the pancreatoscope during manipulation. A small surgical gauze can be placed between the pancreatoscope and the grasper to minimize the damage. The tail side of the gland is first inspected so that frozen section margins can be sent for analysis first to determine if additional resection is necessary. The pancreatic duct is cannulated to the tail end, and the pancreatic duct is examined on withdrawal while flushing the duct with saline, facilitating endoscopic inspection. The upstream gland is then inspected. The pancreatic duct is cannulated until the transition to the duodenum, and the pancreatic duct is similarly inspected on withdrawal (Fig. 1).

### Pancreatoscopy During Robotic Distal Pancreatectomy

During robotic distal pancreatectomy, the superior and inferior borders of the pancreas are dissected at the planned transection site, and the pancreatic gland mobilized off the splenic artery and vein. The pancreatic gland is then encircled with a vessel loop to provide traction. As opposed to pancreatoscopy during a robotic pancreatoduodenectomy when it is performed after the full transection of the pancreatic gland, we perform an anterior ductotomy for the pancreatoscopy during a robotic distal pancreatectomy before its full transection. The hook cautery is used to create the ductotomy to provide access to the pancreatoscope. This ductotomy is performed at a position closer to the spleen to allow sufficient space to identify a good location for parenchymal transection. If the dilated duct is visible externally, a direct ductotomy is performed. In the event of any uncertainty, a drop-in ultrasound transducer can be used to assist with ductal visualization. Care must be taken to not skive beyond the pancreatic duct as the hook cautery can back-wall into the splenic vein posteriorly, and the use of intraoperative ultrasound is advised if the duct is not easily identified. The pancreatoscope can then be passed through either one of the 5–12 mm ports depending on the intra-abdominal angle needed to survey the proximal and distal portions of the pancreatic duct. Either both robotic arms can be used to direct the pancreatoscope into the ductotomy, or one robotic arm used to guide the scope and the other manipulate the pancreatic gland with the vessel loop to provide counter-traction for access. Once the ductal pathology has been identified, a point of parenchymal transection is determined. This is performed by grasping the pancreatoscope at the point of entry in the pancreas once inside the duct when the right location is reached. Then, while maintaining the grasping location on the pancreatoscope, it is placed on top of the pancreas and the transection line is marked using bipolar forceps.

## RESULTS

### Overall Use Robot-assisted Pancreatoscopy

Overall, intraoperative robot-assisted pancreatoscopy was performed in 28 patients undergoing robotic pancreatoduodenectomy (n = 21) and robotic distal pancreatectomy (n = 7) without any serious adverse events or other intraoperative complications resulting from the pancreatoscopy.

### Case Description

There were no intraoperative complications resulting from the intraoperative pancreatoscopy during the robotic pancreatoduodenectomy and robotic distal pancreatectomy presented. The added time required to perform the pancreatoscopy was 16 minutes and 35 seconds during the robotic pancreatoduodenectomy and 6 minutes and 17 seconds during the robotic distal pancreatectomy. No additional resection was needed during the robotic pancreatoduodenectomy as no IPMN was observed in the dilated left-sided main pancreatic duct. This duct was dilated beyond 5 mm but because no villi representing IPMN were observed and the duct appeared fully normal, no further resection was performed.

During the robotic distal pancreatectomy, the pancreatoscopy informed the location of the transection site. This led to less parenchyma being resected than if all of the dilated main pancreatic ducts would have been resected.

Both patients had an uncomplicated hospital course and were discharged on postoperative day 5 after robotic pancreatoduodenectomy and day 6 after robotic distal pancreatectomy.

## DISCUSSION

In this report, we detail the technical approach to intraoperative pancreatoscopy during robotic pancreatoduodenectomy and robotic distal pancreatectomy and highlight tips and tricks to perform this procedure smoothly without an apparent increased risk of complications. The described procedures were performed as part of an international prospective multicenter nonrandomized trial (ClinicalTrials.gov Identifier: NCT03729453)^9^ assessing the added value of pancreatoscopy in patients with IPMN. Publications on robot-assisted pancreatoscopy using the Spy Glass system are lacking and only 1 abstract video report has described pancreatoscopy using a ureteroscope during robotic pancreatoduodenectomy.^9^ Although our anecdotal experience suggests the safety and ease of incorporating pancreatoscopy with robotic pancreatic resections, the final results of the recently completed nonrandomized trial and further prospective studies have to be awaited to assess the intraoperative value and safety of pancreatoscopy on surgical decision-making.
